# Military Medical Rank and Civil Practice

**Published:** 1915-05-01

**Authors:** 


### The Hospital

The Workers' Newspaper of Administrative Medicine and Institutional Life, Administration, National Insurance and Health.

No. 1506, Vol. LVIII. SATURDAY, MAY 1, 1915. PRICE ONE PENNY

### MILITARY MEDICAL RANK AND CIVIL PRACTICE.

Whatever other influence has been exercised by the war on medical practice and the medical profession, the experience of the last nine months has certainly made evident to most people the wide range and essential nature of the services rendered by the R.A.M.C. to the efficiency and success of the fighting ranks. In support of this proposition it is only necessary to quote the "Eye-Witness" who now and again is permitted to tell us from Headquarters something of the intimate life and doings of the Army in the field. The very low general sick-rate of the troops is recognised as mainly due to the preventive measures adopted by the Medical Service, and it is allowed in so many words that but for this Service the Army now fighting on the Continent would certainly have been decimated by disease. All this broad and generous recognition is, of course, welcome to all branches of the profession, and it is perhaps the more grateful to those of us who remember a time when the Army doctors suffered a very different experience, and when a most resolute effort was made to deprive them of any recognised status in the military hierarchy and to reduce them to the position of well-dressed camp followers. Time has worked his revenge, and we may be content, without reviving former bitterness, to respond in all good comradeship to the general and official recognition of medical services as an essential part of the machinery by which the nation's honour and the nation's interest are maintained. Whatever criticism may have been heard at other times, no one just now, we imagine, is desirous of taking any step which would either in form or substance separate the status of the Army doctor from that of his brother officers. It is true that in one of our lay weekly contemporaries Mr. Stephen Coleridge judges the time appropriate to indulge in some sneering remarks on certain medical activities and opinions, and in this respect he does not belie his record. But from the men in the trenches, as well as from those who are immediately concerned with the Army's welfare, we hear nothing but appreciation and gratitude; and in these circumstances sarcasms elaborated in a comfortable country house need hardly disturb our peace.

It would seem that, apart altogether from the claims of justice and sentiment, the necessity for efficient administration and successful discipline make it advisable, or even essential, that the Army doctors should have military rank. They are part of a complicated organisation in which the relative position of the various grades is a prominent part of the scheme; and he who has not his place so fixed and determined is likely to lack both recognition and authority. What is true of the Regular Army is true no doubt also of Territorial troops when these are called on to take the field, and here, therefore, the doctors have their military titles and rank. Upon mobilisation, and to the surprise of many people, there appeared another group of Army doctors, men whose attitude and characters up to that moment had been purely those of the peaceful citizen. We refer, of course, to the holders of the *à la suite* commissions. These gentlemen, mostly hospital physicians or surgeons, suddenly blossomed out as lieutenant-colonels, majors, captains, and so on, and were appointed as visiting medical officers of various military hospitals. Here, again, the suggestion is that the demands of discipline make the adoption of uniform and rank a necessity of the position, that without these aids the wounded soldier will not accept orders nor can the medical officers themselves be efficiently directed and controlled.

With the arrangements as here sketched in general terms we have no desire to quarrel. If the Army authorities say they are necessary to the quiet working of their machine we are willing, at least in the meantime, to concur. We merely add to our acquiescence a note that the adoption of military titles by the doctors is a step taken at the wish of the Army authorities and in the interests of Army discipline. To be called a captain or colonel means for a physician or surgeon no increase either of professional status or personal dignity, and most doctors, we fancy, would prefer to avoid these titles. Further, under pressure, we are glad to believe that the War Office has come to see that medical and surgical services can, at least in certain circumstances, be rendered to the Army by practitioners who preserve both the titles and duties of civil life. It is, we understand, a well-founded report that a number of physicians and surgeons in London, and having no military associations, have, in response to Sir Alfred Keogh's appeal, undertaken to staff the further military hospitals which the War Office is shortly to establish. One of the conditions of this offer is that those making it shall not be expected to take commissions in the R.A.M.C., but shall remain free to attend to the hospital and other duties for which they are at present responsible. We venture to express our entire sympathy with these conditions and our hope that they will be firmly maintained. Two things are certain: One, that many medical men who at present hold military commissions are still left with time on their hands; and the other that our civil hospitals must be carried on in the interests alike of humanity and of the national welfare. The new proposals are based upon a recognition of these two observations, and they seem to us to be characterised by an eminently reasonable and common sense spirit and by a broad and healthy view of the facts of the situation.

